# Marker-assisted pyramiding of γ-tocopherol methyltransferase and glutamate formiminotransferase genes for development of biofortified sweet corn hybrids

**DOI:** 10.7717/peerj.13629

**Published:** 2022-07-06

**Authors:** Guihua Lv, Xiaolong Chen, Duo Ying, Jiansheng Li, Yinghu Fan, Bin Wang, Ruiqiu Fang

**Affiliations:** 1 Institute of Maize and Featured Upland Crops, Zhejiang Academy of Agricultural Sciences, Dongyang, Zhejiang, China; 2 National Maize Improvement Center of China, Beijing Key Laboratory of Crop Genetic Improvement, China Agricultural University, Beijing, China; 3 Chuxiong Academy of Agricultural Sciences, Chuxiong, China; 4 Institute of Vegetables, Zhejiang Academy of Agricultural Sciences, Hangzhou, Zhejiang, China

**Keywords:** Biofortification, Hybrids, Molecular breeding, Sweet corn

## Abstract

Micronutrients, including vitamins, minerals, and other bioactive compounds, have tremendous impacts on human health. Much progress has been made in improving the micronutrient content of inbred lines in various crops through biofortified breeding. However, biofortified breeding still falls short for the rapid generation of high-yielding hybrids rich in multiple micronutrients. Here, we bred multi-biofortified sweet corn hybrids efficiently through marker-assisted selection. Screening by molecular markers for vitamin E and folic acid, we obtained 15 inbred lines carrying favorable alleles (six for vitamin E, nine for folic acid, and three for both). Multiple biofortified corn hybrids were developed through crossing and genetic diversity analysis.

## Introduction

Micronutrients (vitamins and minerals) are essential to people’s health (Farré et al., 2014). At present, billions of people (mainly in developing countries) still suffer from “hidden hunger” due to insufficient intake of micronutrients (Muthayya et al., 2013). In the past 20 years, biofortification, enhancement of the levels of micronutrients in food crops through agricultural technologies, has been used as an important strategy to produce healthier food (Garg et al., 2018; Saltzman et al., 2017).

Sweet corn (*Zea mays* L. var. saccharata), a type of maize with high levels of sugar, is an invaluable source of protein, calories, essential fatty acids, vitamins, and minerals for human nutrition (Wu et al., 2020). However, the content of micronutrients present in the different sweet corn varieties varied significantly. The wide variability for micronutrient content in sweet corn unveils the great prospect of developing biofortified sweet corn varieties. Many quantitative trait loci (QTL) associated with micronutrients content have been identified (Baseggio et al., 2019; Baseggio et al., 2020; Diepenbrock et al., 2017; Hershberger et al., 2021; Lone et al., 2021; Simic et al., 2012; Wu et al., 2022). For example, *ZmVTE4*, encoding γ-tocopherol methyltransferase, is capable of catalyzing γ-tocopherol to α-tocopherol. α-tocopherol, the major constituent of vitamin E, shows the highest vitamin E activity (Burton & Ingold, 1981; Kamal-Eldin & Appelqvist, 1996). Two insertions in *ZmVTE4* promoter region and 5′ untranslated region (5′ UTR) affect the level of α-tocopherol through regulating gene expression (Li et al., 2012). Molecular markers (InDel7 and InDel118) corresponding to the two insertions were developed to screen for the favorable alleles. ZmCTM (catalysis of 5-M-THF to MeFox) functions as a key enzyme to convert 5-methyl-tetrahydrofolate (5-M-THF) to a pyrazino-s-triazine derivative of 4 α-hydroxy-5-methyl-tetrahydrofolate (MeFox) in folate metabolism. MeFox is the stable storage form of folic acid in seeds (Goyer et al., 2005). The natural asparagine-to-glycine substitution caused by an A-to-G single nucleotide variation in *ZmCTM* coding region enhances its enzymatic activity (Zhang et al., 2016). The G-allele can be identified by marker SNP682.

Commercial seeds of sweet corn are mostly F_1_ hybrids, which are phenotypically superior and with significantly higher yield compared to their parents. Traditional corn breeding based on genetic crosses requires identifying the best parental combinations for creating elite hybrids. This process is very laborious, time-consuming, and cost ineffective. Moreover, the results are usually unpredictable and not always accurate. The level of genetic diversity between two parents has been proposed as a possible predictor of F_1_ performance in crops (Yousuf et al., 2021). Accurate characterization of the genetic background of inbred lines can be very useful in selecting inbred lines for crossing (Beckett et al., 2017). The genetic variability can be assessed using agro-morphological traits, which may result in misleading estimates due to higher influence of environment on them. With the development of functional genomics and genome sequencing, marker-assisted selection has become an important approach for current crop improvement (Nie et al., 2014). Previous study established a core set of SSR molecular marker for characterizing genetic diversity of Chinese maize varieties and establishing the identity of new varieties (Wang et al., 2011).

Impressive progress has been made in biofortification of different elite crop inbred lines (Prasanna et al., 2019). There is an increasing demand for hybrid lines in practical production. Based on these requirements, we wondered whether genetic diversity together with favorable allele for vitamin E and/or folic acid could be analyzed to develop multi-biofortified sweet corns hybrids. Here, we obtained 15 inbred lines carrying favorable alleles through screening by molecular marker for vitamin E and folic acid (Li et al., 2012; Zhang et al., 2016). Together with the genetic diversity analysis (Wang et al., 2011), multiple biofortified corn hybrids were developed through crossing. This approach should greatly accelerate future biofortified breeding of sweet corn hybrids via effective selection of elite inbred lines with biofortification traits suit for optimal combination.

## Materials and Methods

### Plant material

A set of 52 sweet corn inbred lines procured from different sources and maintained through selfing were taken for the study (Table S1). All these inbreds were planted in a randomized block design with two replications at the farmland of Zhejiang Academy of Agricultural Sciences (Dongyang, China) during 2020 and 2021.

### Genetic diversity analysis and allele screening

Genomic DNA was extracted using a modified CTAB extraction protocol (Clarke, Moran & Appels, 1989). The core 40 SSR primers were used for genetic diversity analysis (Table S2) (Wang et al., 2011). PCR amplifications were performed with a final reaction volume of 20 µL containing 30∼40 ng genomic DNA. The PCR conditions were: 94 °C for 2 min, followed by 35 cycles of denaturation at 94 °C for 30 s, annealing at 55 °C for 30 s, extension at 72 °C for 30 s, and a last extension step at 72 °C for 10 minutes. The amplified products were resolved using 1.5% agarose gel or 12% PAGE (polyacrylamide gel electrophoresis) gel. Calculation of the PIC (polymorphism information content value) was based on the results obtained from SSR using the following formula: PIC = 1−Σfi^2^, where fi^2^ isthe frequency of the allele. A dendrogram were created using the unweighted pair group method using arithmetic averages (UPGMA) feature of NTSYS-pc software Version 2.2. InDel7 and InDel118 were used for *ZmVTE4* allele screening (Li et al., 2012), SNP682 was used for *ZmCTM* allele screening (Table 1) (Zhang et al., 2016). Primer sequences were obtained from the previously published paper by Li et al. (2012) and Zhang et al. (2016) with minor modifications.

### Quantification of free α-tocopherol

The endogenous free α-tocopherol contents were determined by Wuhan Greensword Creation Technology Co. Ltd. (Wuhan, China) based on UHPLC-MS/MS analysis. In brief, sample were frozen in liquid nitrogen, ground to fine powder, and extracted with 1.0 mL n-hexane at −20 °C for 12 h. After centrifugation (10,000 g, 4 °C, 20 min), the supernatants were collected and evaporated under mild nitrogen stream at 35 °C followed by re-dissolving in 100 µL ACN for UHPLC-MS/MS analysis (Thermo Scientific Ultimate 3000 UHPLC coupled with TSQ Quantiva; Thermo Fisher Scientific, Waltham, MA).

### Quantification of free folic acid

The endogenous free folic acid contents were determined by Wuhan Greensword Creation Technology Co. Ltd. (Wuhan, China) based on UHPLC-MS/MS analysis. In brief, sample were frozen in liquid nitrogen, ground to fine powder, and extracted with 1.0 mL 80% methanol aqueous solution at −20 °C for 12 h. After centrifugation (10,000 g, 4 °C, 20 min), the supernatants were collected and evaporated under mild nitrogen stream at 35 °C followed by re-dissolving in 100 µL 50% ACN for UHPLC-MS/MS analysis (Thermo Scientific Ultimate 3000 UHPLC coupled with TSQ Quantiva; Thermo Fisher Scientific, Waltham, MA).

**Table 1:** Primers for InDel7, InDel118, and SNP682 used in this study.

**Gene**	**Polymorphic site**	**Prime direction**	**Primer sequences (5′-3′)**
*ZmCTM*	ZmCTM-CDS	Forward	TACGACGGTGGGTGTCAC
		Reward	TGATAGGCGCTGGCATGATC
	ZmCTM-CDS2	Forward	GTCATGCCTTGGATCGTGGG
		Reward	ATGACGTCCTTACACAGCAC
*ZmVTE4*	ZmVTE4-InDel7	Forward	TGCCGGCACCTCTACTTTAT
		Reward	AGGACTGGGAGCAATGGAG
*ZmVTE4*	ZmVTE4-InDel118	Forward	AAAGCACTTACATCATGGGAAAC
		Reward	TTGGTGTAGCTCCGATTTGG

## Results and Discussion

To test the feasibility of the strategy, we analyzed the genetic diversity of 52 widely used sweet corn inbred lines using 40 pairs of SSR core markers (Wang et al., 2011). These markers produced 226 alleles, an average of 5.7 alleles per marker, suggesting a high frequency of allelic variation. The value of polymorphism information content (PIC) for each SSR locus varied between 0.27 and 0.87 with an average of 0.60. Based on the classification of PIC (PIC value < 0.25, low; 0.25 < PIC value < 0.5, intermediate; and PIC value > 0.5, high polymorphism) (Botstein et al., 1980), all the 40 SSR makers were found with moderate polymorphism and heterozygosity. The results suggested that these 40 SSR markers are suitable for assessing genetic diversity of sweet corn resources.

**Figure 1:**
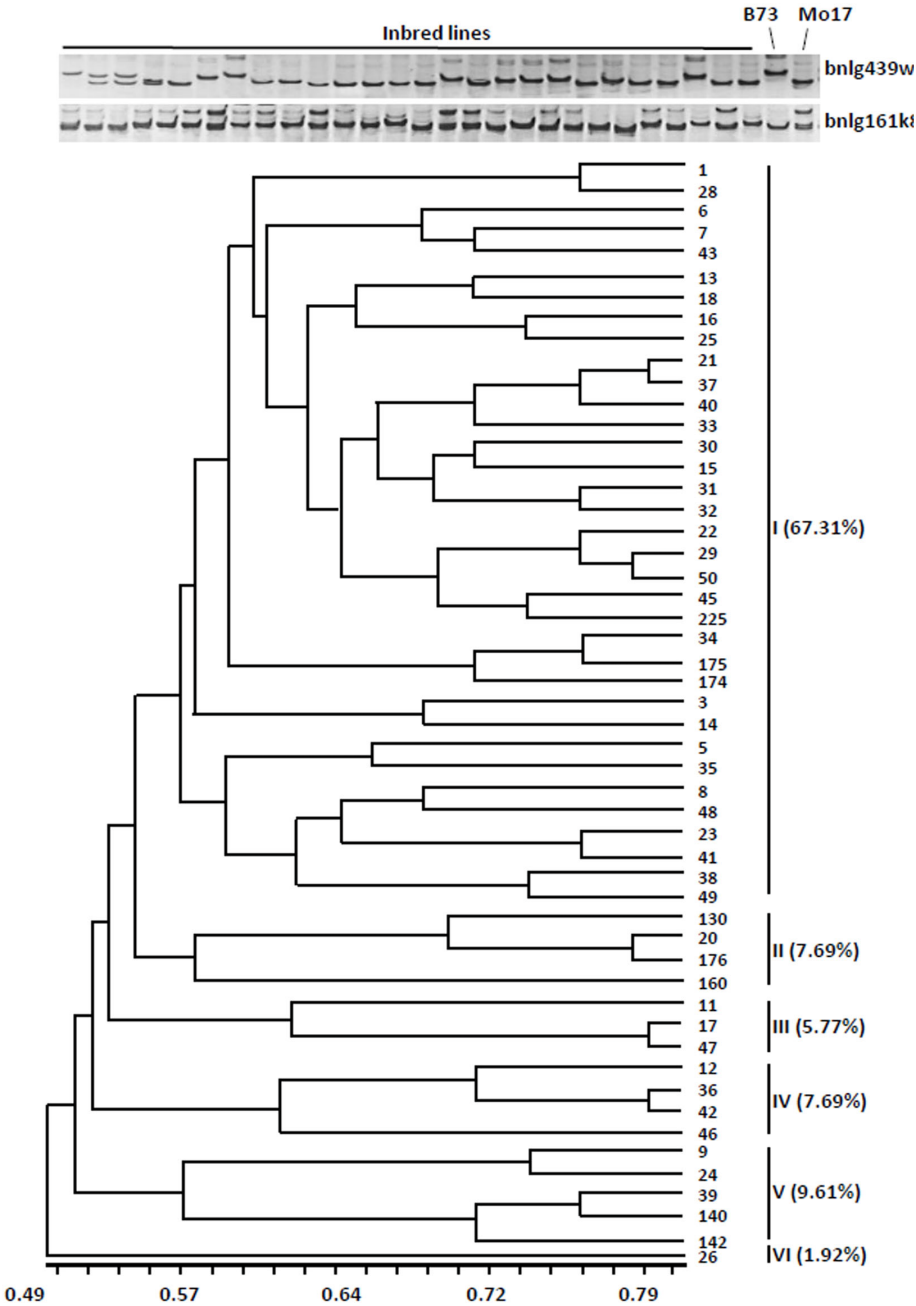
Cluster dendrogram depicting genetic divergence among 52 inbreds based on 40 core molecular markers. (A) Microsatellite polymorphism among sweet corn inbreds. (B) Cluster dendrogram depicting genetic divergence among 52 inbreds based on 40 core molecular markers.

The dendrogram was obtained from the similarity coefficient and clustering was done by using the UPGMA algorithm with the NTSYS software program. The 52 inbred lines were divided into six distinct groups at the similarity coefficient level of 0.55 (Fig. 1). The first group accounted for 67.31% (35 inbred lines), the other groups were only for 32% (17 inbred lines). These results indicated that most of the inbred lines have similar genetic background.

**Figure 2:**
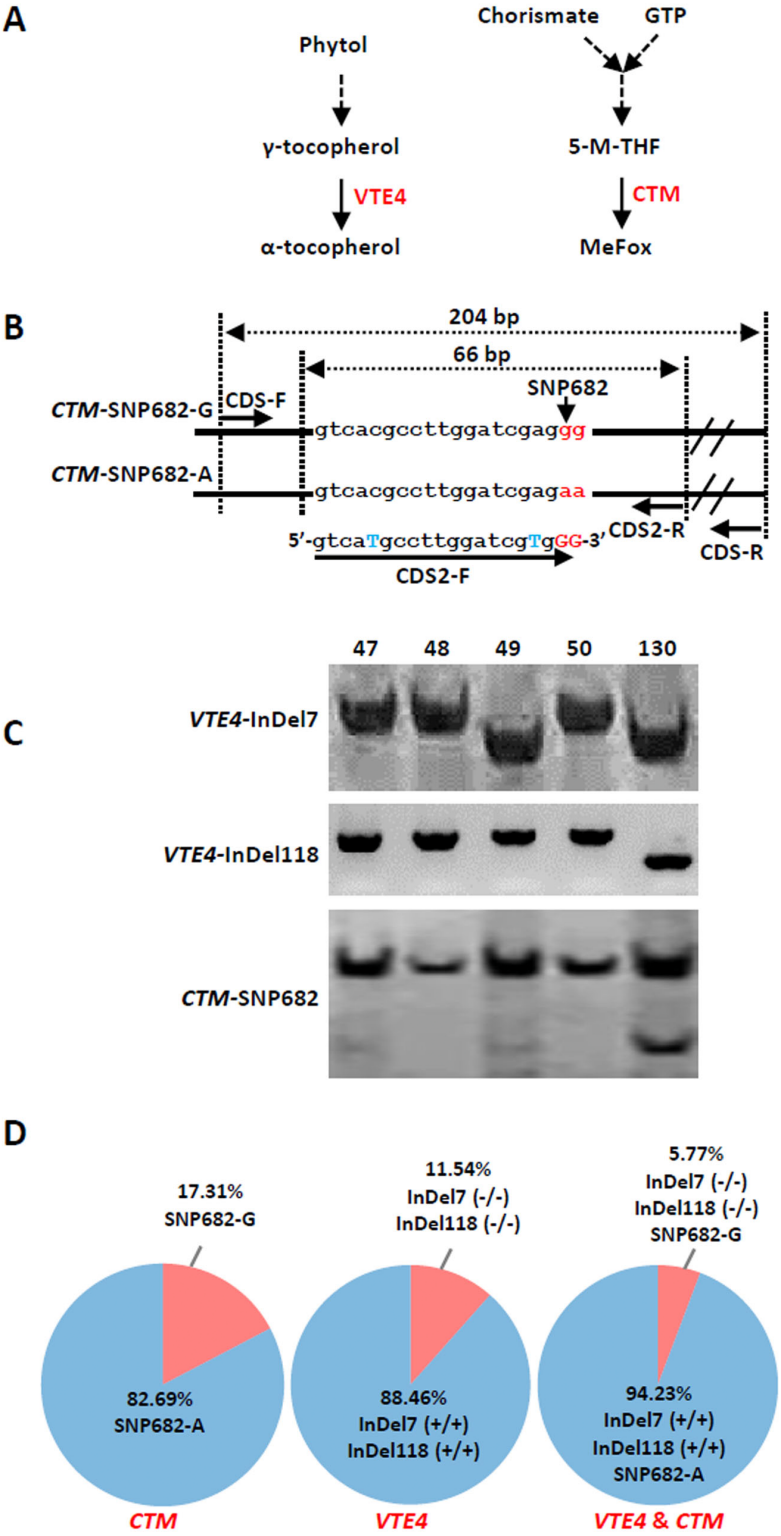
Screening of favorable alleles for vitamin E and/or folic acid in the 52 inbred lines. (A) Schematic of α-tocopherol and folate metabolism. VTE4, *γ*-tocopherol methyltransferase; 5-M-THF, 5-methyl-tetrahydrofolate; MeFox, a pyrazino-s-triazine derivative of 4 α-hydroxy-5-methyl-tetrahydrofolate; CTM, catalysis from 5-M-THF to MeFox. (B) Schematic illustration of SNP682 loci primer design, blue upper-case letters represent bases substituted to balance primer GC content of primer. (C) Representative pictures of allele assay at InDel7, InDel118, and SNP682 loci. (D) Analysis of allele at InDel7, InDel118, and SNP682 loci among 52 inbreds. InDel7 (+/ +), homozygous 7-bp insertion in the 5′ untranslated region (5′ UTR) of *ZmVTE4*; InDel7 (−/−), homozygous 0-bp insertion in the 5′ untranslated region (5′ UTR) of *ZmVTE4*; InDel118 (+/+), homozygous 118-bp insertion in the promoter region of *ZmVTE4*; InDel118 (−/−), homozygous 0-bp insertion in the promoter region of *ZmVTE4*; SNP682-G, homozygous G at position 682 in the coding sequence of *ZmCTM*; SNP682-A, homozygous A at position 682 in the coding sequence of *ZmCTM*.

We further analyzed the favorable alleles associated with vitamin E and folic acid content in 52 sweet corn inbred lines. ZmVTE4 and ZmCTM were identified to regulate biosynthesis of free α-tocopherol and folic acid content, respectively (Fig. 2A). Molecular markers (InDel7 and InDel118) corresponding to the two insertions in *ZmVTE4* promoter region and 5′ untranslated region (5′ UTR) were used to screen favorable allele for free α-tocopherol. Marker SNP682 in *ZmCTM* coding region was used to characterize alleles for free folic acid. Genotypic screening showed that there was 11.54% (*n* = 6) lines with deletion-allele in InDel7 and InDel118 loci, 17.31% (*n* = 9) lines with G-allele in SNP682 and 5.77% (*n* = 3) lines with both deletion-allele and G-allele (Fig. 2D). Our results revealed that most of the elite inbred lines used in breeding do not contain favorable alleles associated with vitamin E and folic acid content.

**Figure 3:**
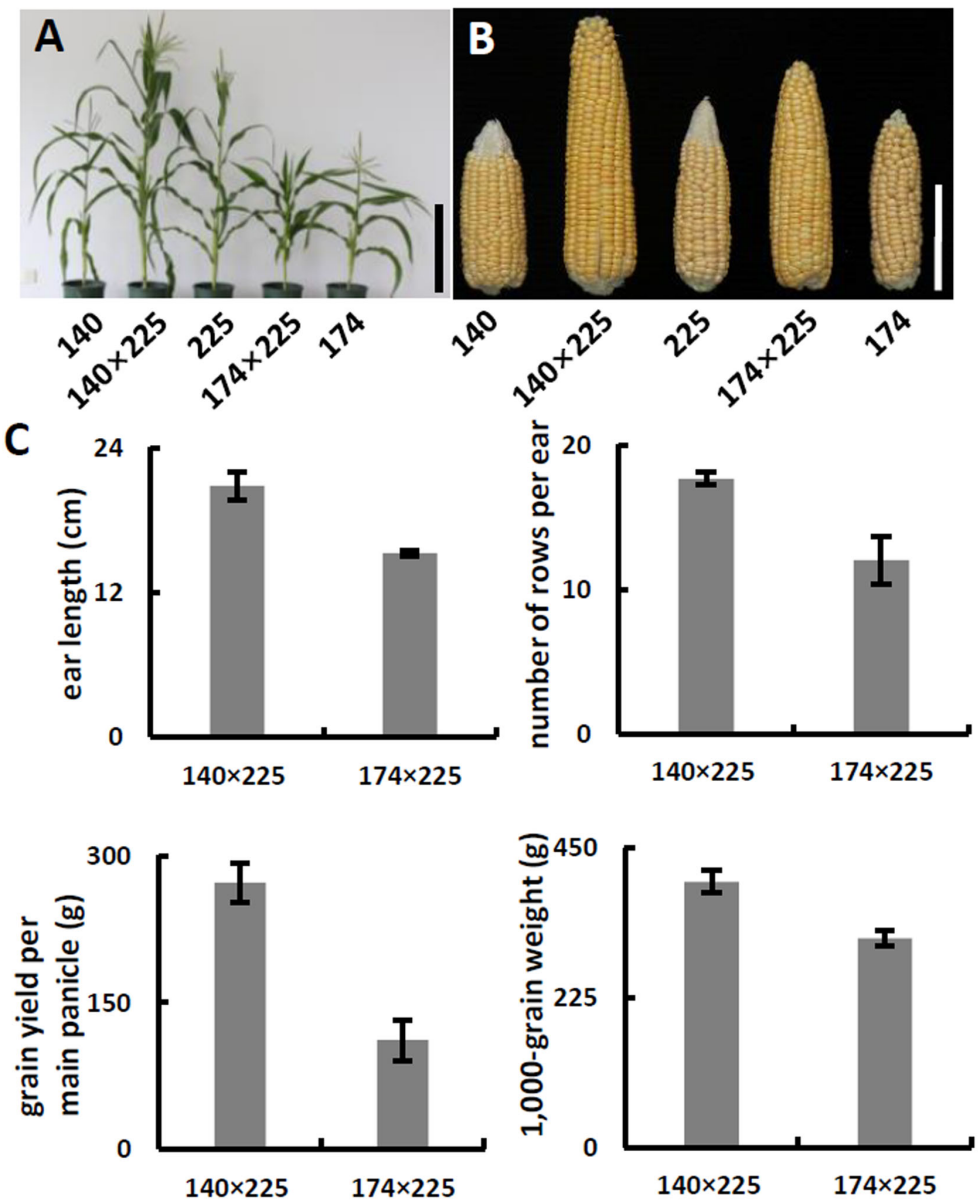
Phenotypes and agronomic traits of parental inbreds and hybrids. (A) Plant phenotype of parental inbreds and hybrids. Bar, 30 cm. (B) Phenotype of parental inbreds and hybrids on ears. Bars, 10 cm. (C) Analysis of agronomic traits hybrid 140 × 225 and hybrid 174 × 225. Error bars represent s.d.

To develop hybrids with high level of vitamin E and folic acid, we chose inbred lines with micronutrients associated favorable alleles for crossing. Previous studies have suggested that the level of genetic diversity between two parents could be used as a possible predictor of F_1_ performance in crops (Xiao et al., 1996). Among the inbred lines carrying favorable alleles associated with vitamin E and folic acid content, lines with different genetic distance were selected to cross as parents. F_1_ progenies from lines crosses with a large genetic distance (140 × 225, 142 × 225, 140 × 15, and 142 × 15) were observed with favorable agronomic traits (ear length, number of rows per ear, grain yield per main panicle, and 1,000-grain weight) (Figs. 3A–3C, Table 2). Notably, the highest yield per plant (272.58 g) was hybrid 140 × 225. In contrast, F_1_ progenies of lines from same group had poor agronomic traits (Figs. 3A–3C, Table 2). The same trend can be found for other hybridization combination.

**Figure 4:**
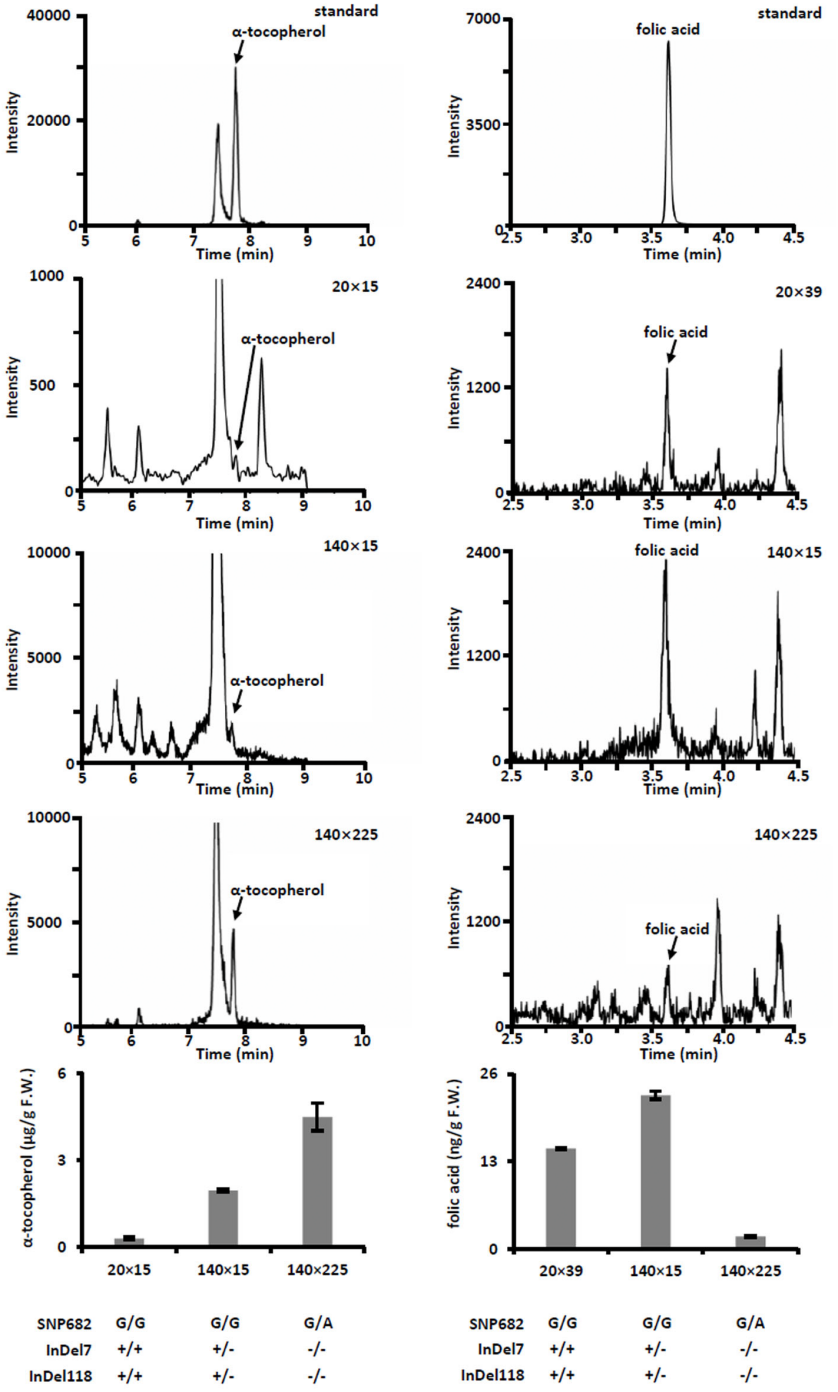
Quantification of free α-tocopherol and folic acid in kernel of hybrids. Error bars represent s.d.

Meanwhile, we measured free α-tocopherol and folic acid in F_1_ progenies. Based on the allele analysis, we found that hybrid 140 × 15 and hybrid 140 × 225 contains α-tocopherol favorable allele (InDel7^+/−^InDel118^+/−^ for hybrid 140 × 15 and InDel7^−/−^InDel118^−/−^for hybrid 140 × 225). Insertion in InDel7 and InDel118 loci affect the expression of *ZmVTE4* (Li et al., 2012). Quantification of free α-tocopherol (main component of vitamin E) revealed that the concentration in hybrid 140 × 15 and hybrid 140 × 225 was lower than that in hybrid 20 × 15 carrying no α-tocopherol favorable allele (InDel7^+/+^InDel118^+/+^) (Fig. 4). A similar variation pattern was observed for free folic acid in sweet corn kernel. The asparagine-to-glycine substitution caused by an A-to-G single nucleotide variation (SNP682) in maize *ZmCTM* enhances its enzymatic activity (Zhang et al., 2016). Homozygous G (SNP682^G/G^) carrying hybrid 140 × 15 and 20 × 39 had significantly higher levels of free folic acid than heterozygous G/A (SNP682^G/A^) carrying hybrid 140 × 15 in kernel (Fig. 4). In addition, there are differences between hybrid 140 × 15 and 20 × 39. Folates are unstable compounds, susceptible to oxidative and photo-oxidative catabolism (Blancquaert et al., 2014). Vitamin E is a potent antioxidant in plants, widely used to increase the shelf life of β-carotene in foods (Choe & Min, 2009). High level of α-tocopherol in Hybrid 140 × 15 may enhance folate stability. Our results demonstrated the validity of the strategy and provided supporting evidence for the notion that *ZmVTE4* (Li et al., 2012) and *ZmCTM* (Zhang et al., 2016) are key for the regulation of vitamin E and folic acid level in maize kernel.

**Table 2:** Characterization of agronomic traits of hybrids. Note, different letters show significant differences among treatment combinations at 5 probability level using Duncans multiple range test.

**Hybrid F1**	**Growth phases (days)**	**Plant height** **(m)**	**Ear length** **(cm)**	**Number of rows per ear**	**1,00-grain weight (g)**	**Grain yield per main panicle (g)**	**Sucrose content (mg/g)**
15 × 20	86	1.75 ± 4.24c	16.17 ± 0.97gh	15 ± 2.16abc	47.27 ± 3.96a	203.77 ± 25.1bcd	172.39 ± 17.72b
15 × 28	90	2.38 ± 0.02a	19.77 ± 0.33bc	15 ± 0.82abc	34.31 ± 1.85de	245.79 ± 7.49cd	148.59 ± 13.72bcde
20 × 39	86	1.65 ± 1.25cd	18.23 ± 0.63def	16.67 ± 2.49ab	48.59 ± 2.24a	231.24 ± 36.95abc	118.53 ± 24.05
140 × 15	92	2.32 ± 0.02ab	20.73 ± 0.45ab	15.67 ± 0.47abc	45.04 ± 2.85a	263.29 ± 24.72a	126.18 ± 0.97ef
142 × 15	92	2.28 ± 0.09ab	18.97 ± 0.92cde	17.33 ± 1.25ab	35.22 ± 0.3cde	199.14 ± 9.95bcd	140.8 ± 7.82def
140 × 142	89	2.36 ± 0.06a	17.5 ± 0.82efg	14 ± 2.83abc	36.11 ± 1.06bcd	158.08 ± 13.49de	144 ± 15.49cde
140 × 174	88	2.28 ± 0.07ab	17.93 ± 0.19def	14.67 ± 0.47abc	33.23 ± 0.74de	159.11 ± 5.27de	173.94 ± 4.33b
174 × 175	87	1.69 ± 0.03cd	17.4 ± 0.38fg	15.33 ± 0.47abc	34.31 ± 0.85de	172 ± 10.71d	172.55 ± 3.51b
142 × 175	91	2.22 ± 0.04b	19.37 ± 0.7bcd	18.67 ± 0.47a	33.36 ± 1.58de	232.2 ± 11.52abc	157.06 ± 4.85bcd
39 × 225	89	2.34 ± 0.07a	21.77 ± 0.39a	17 ± 2.16ab	38.92 ± 0.47bc	261.75 ± 20.23a	159.05 ± 7.84bcd
140 × 225	89	2.31 ± 0.01ab	20.83 ± 1.19ab	17.67 ± 0.47ab	39.92 ± 1.68b	272.58 ± 20.23a	167.1 ± 5.19bc
142 × 225	90	2.28 ± 0.04ab	21.4 ± 0.43a	16.67 ± 1.7ab	36.55 ± 1.3bcd	237.5 ± 41.05ab	163.12 ± 2.65bcd
174 × 225	92	1.59 ± 0.03d	15.23 ± 0.21h	12 ± 1.63c	31.41 ± 1.23e	111.29 ± 20.79e	205.52 ± 3.79a

## Conclusion

It is known that molecular marker-assisted selection is used in crop breeding (Jena & Mackill, 2008). Given that most commercial seeds are hybrids, we envisage that the strategy used here will be widely adopted to accelerate biofortification breeding of various crops. The strategy allows for biofortification in elite F_1_ hybrid with much higher efficiency and accuracy. A further improvement of this strategy could be achieved by integrating morphological traits assay to characterize genetic structure of parent lines comprehensively (Mahato et al., 2018). Further, the development of new polymorphic detection technologies such as KASP (Semagn et al., 2014), and whole-genome resequencing (Jiao et al., 2012; Mace et al., 2013) would also greatly expand the utility of this strategy. The strategy described here hold great promise to future biofortification breeding.

## Supplemental Information

10.7717/peerj.13629/supp-1 Supplemental Information 1 Raw data for Figure 3 and Table 2

10.7717/peerj.13629/supp-2 Supplemental Information 2 Raw data for Figure 4

10.7717/peerj.13629/supp-3 Supplemental Information 3 Supplemental Table S1 List of sweet corn inbred lines. Supplemental Table S2 Details of markers used for genetic diversity analysis

## Additional Information and Declarations

### Data Availability

The following information was supplied regarding data availability: The raw measurements are available in the Supplementary Files.
