# Supplementary material for: Marker-assisted pyramiding of γ-tocopherol methyltransferase and glutamate formiminotransferase genes for development of biofortified sweet corn hybrids
Source: PeerJ. 2022 Jul 6;10:e13629. doi: 10.7717/peerj.13629 (PMC9270877; doi:10.7717/peerj.13629)
Supplement: Supplemental Information 3 [file peerj-10-13629-s003.docx]

**Supplemental Table S1 List of sweet corn inbred lines.**

**Supplemental Table S2 Details of markers used for genetic diversity analysis.**

**Table S1 List of sweet corn inbred lines.**

| **No.** | **Inbred line** | **No.** | **Inbred line** | **No.** | **Inbred line** |
| --- | --- | --- | --- | --- | --- |
| 1 | WT2-1 | 23 | 2018i | 42 | DA28-2 |
| 3 | 2017i126 | 24 | X5-211 | 43 | G17/A218 |
| 5 | 2017i55 | 25 | H06 | 45 | 17i1221 |
| 6 | DU-193 | 26 | X5M | 46 | 187 |
| 7 | 2017i20 | 28 | X5F | 47 | 218 |
| 8 | 2018-2 | 29 | TJNKY1372 | 48 | K11 |
| 9 | XT7401-19 | 30 | T521 | 49 | 15i151 |
| 11 | HNH1401 | 31 | 17hi2550 | 50 | MF |
| 12 | HNB1301 | 32 | JQC | 130 | SS4 |
| 13 | 901 | 33 | 2476 | 140 | JH8 |
| 14 | Z6A8-366 | 34 | 2450 | 142 | JH7 |
| 15 | 12hi307 | 35 | TN6 | 160 | 17i132 |
| 16 | H2-3 | 36 | W3000 | 174 | JP131 |
| 17 | Jg218 | 37 | W11 | 175 | 2017i246 |
| 18 | 1014 | 38 | HT-4 | 176 | 15D |
| 20 | M688 | 39 | 2558 | 225 | B2H18 |
| 21 | D6 | 40 | 2525 |  |  |
| 22 | T15 | 41 | 18iYGHF-001 |  |  |

**Table S2 Details of markers used for genetic diversity analysis.**

| **Loci name** | **Motif sequence** | **Forward primer sequence (5’-3’)** | **Reverse primer sequence (5’-3’)** |
| --- | --- | --- | --- |
| bnlg439w1 | (TC) | AGTTGACATCGCCATCTTGGTGAC | GAACAAGCCCTTAGCGGGTTGTC |
| umc1335y5 | (AG) | CCTCGTTACGGTTACGCTGCTG | GATGACCCCGCTTACTTCGTTTATG |
| umc2007y4 | (TC) | TTACACAACGCAACACGAGGC | GCTATAGGCCGTAGCTTGGTAGACAC |
| bnlg1940k7 | (CT) | CGTTTAAGAACGGTTGATTGCATTCC | GCCTTTATTTCTCCCTTGCTTGCC |
| umc2105k3 | (AG) | GAAGGGCAATGAATAGAGCCATGAG | ATGGACTCTGTGCGACTTGTACCG |
| phi053k2 | (GTAT) | CCCTGCCTCTCAGATTCAGAGATTG | TAGGCTGGCTGGAAGTTTGTTGC |
| phi072k4 | (TGTT) | GCTCGTCTCCTCCAGGTCAGG | CGTTGCCCATACATCATGCCTC |
| bnlg2291k4 | (AG) | GCACACCCGTAGTAGCTGAGACTTG | CATAACCTTGCCTCCCAAACCC |
| umc1705w1 | (CT) | GGAGGTCGTCAGATGGAGTTCG | CACGTACGGCAATGCAGACAAG |
| bnlg2305k4 | (GA) | CCCCTCTTCCTCAGCACCTTG | CGTCTTGTCTCCGTCCGTGTG |
| bnlg161k8 | (AG) | TCTCAGCTCCTGCTTATTGCTTTCG | GATGGATGGAGCATGAGCTTGC |
| bnlg1702k1 | (CT) | GATCCGCATTGTCAAATGACCAC | AGGACACGCCATCGTCATCA |
| umc1545y2 | (AAGA) | AATGCCGTTATCATGCGATGC | GCTTGCTGCTTCTTGAATTGCGT |
| umc1125y3 | (CTCG) | GGATGATGGCGAGGATGATGTC | CCACCAACCCATACCCATACCAG |
| bnlg240k1 | (GA) | GCAGGTGTCGGGGATTTTCTC | GGAACTGAAGAACAGAAGGCATTGATAC |
| phi080k15 | (GGAGA) | TGAACCACCCGATGCAACTTG | TTGATGGGCACGATCTCGTAGTC |
| phi065k9 | (GTGAA/GTGCA) | CGCCTTCAAGAATATCCTTGTGCC | GGACCCAGACCAGGTTCCACC |
| umc1492y13 | (GCA) | GCGGAAGAGTAGTCGTAGGGCTAGTGTAG | AACCAAGTTCTTCAGACGCTTCAGG |
| umc1432y6 | (TC) | GAGAAATCAAGAGGTGCGAGCATC | GGCCATGATACAGCAAGAAATGATAAGC |
| umc1506k12 | (TTTG) | GAGGAATGATGTCCGCGAAGAAG | TTCAGTCGAGCGCCCAACAC |
| umc1147y4 | (CA) | AAGAACAGGACTACATGAGGTGCGATAC | GTTTCCTATGGTACAGTTCTCCCTCGC |
| bnlg1671y17 | (CT) | CCCGACACCTGAGTTGACCTG | CTGGAGGGTGAAACAAGAGCAATG |
| phi96100y1 | (AGGT) | TTTTGCACGAGCCATCGTATAACG | CCATCTGCTGATCCGAATACCC |
| umc1536k9 | (GT/TA) | TGATAGGTAGTTAGCATATCCCTGGTATCG | GAGCATAGAAAAAGTTGAGGTTAATATGGAGC |
| bnlg1520K1 | (CT/AC/GA/TA) | CACTCTCCCTCTAAAATATCAGACAACACC | GCTTCTGCTGCTGTTTTGTTCTTG |
| umc1489y3 | (GCG) | GCTACCCGCAACCAAGAACTCTTC | GCCTACTCTTGCCGTTTTACTCCTGT |
| bnlg490y4 | (TA) | GGTGTTGGAGTCGCTGGGAAAG | TTCTCAGCCAGTGCCAGCTCTTATTA |
| umc1999y3 | (TGC) | GGCCACGTTATTGCTCATTTGC | GCAACAACAAATGGGATCTCCG |
| umc2115k3 | (GCCAT) | GCACTGGCAACTGTACCCATCG | GGGTTTCACCAACGGGGATAGG |
| umc1429y7 | (AGC) | CTTCTCCTCGGCATCATCCAAAC | GGTGGCCCTGTTAATCCTCATCTG |
| bnlg249k2 | (AG) | GGCAACGGCAATAATCCACAAG | CATCGGCGTTGATTTCGTCAG |
| phi299852y2 | (CTG) | AGCAAGCAGTAGGTGGAGGAAGG | AGCTGTTGTGGCTCTTTGCCTGT |
| umc2160k3 | (AG) | TCATTCCCAGAGTGCCTTAACACTG | CTGTGCTCGTGCTTCTCTCTGAGTATT |
| umc1936k4 | (TG) | GCTTGAGGCGGTTGAGGTATGAG | TGCACAGAATAAACATAGGTAGGTCAGGTC |
| bnlg2235y5 | (TG) | CGCACGGCACGATAGAGGTG | AACTGCTTGCCACTGGTACGGTCT |
| phi233376y1 | (CCG) | CCGGCAGTCGATTACTCCACG | CAGTAGCCCCTCAAGCAAAACATTC |
| umc2084w2 | (CTAG) | ACTGATCGCGACGAGTTAATTCAAAC | TACCGAAGAACAACGTCATTTCAGC |
| umc1231k4 | (GA) | ACAGAGGAACGACGGGACCAAT | GGCACTCAGCAAAGAGCCAAATTC |
| phi041y6 | (CAGC) | CAGCGCCGCAAACTTGGTT | TGGACGCGAACCAGAAACAGAC |
| umc2163w3 | (AG) | CAAGCGGGAATCTGAATCTTTGTTC | CTTCGTACCATCTTCCCTACTTCATTGC |
